# A large animal model for standardized testing of bone regeneration strategies

**DOI:** 10.1186/s12917-018-1648-0

**Published:** 2018-11-06

**Authors:** James C. Ferguson, Stefan Tangl, Dirk Barnewitz, Antje Genzel, Patrick Heimel, Veronika Hruschka, Heinz Redl, Thomas Nau

**Affiliations:** 1 Ludwig Boltzmann Institute for Experimental and Clinical Traumatology in AUVA research center, Vienna, Austria; 2 Austrian Cluster for Tissue Regeneration, Vienna, Austria; 3 Department of Evolutionary Anthropology, Faculty of Life Sciences, University of Vienna, Vienna, Austria; 4 Karl Donath Laboratory for Hard Tissue and Biomaterial Research, Department of Oral Surgery, School of Dentistry, Medical University of Vienna, Vienna, Austria; 5 Research Centre of Medical Technology and Biotechnology, fzmb GmbH, Bad Langensalza, Germany

## Abstract

**Background:**

The need for bone graft substitutes including those being developed to be applied together with new strategies of bone regeneration such as tissue engineering and cell-based approaches is growing. No large animal model of bone regeneration has been accepted as a standard testing model. Standardization may be the key to moving systematically towards better bone regeneration. This study aimed to establish a model of bone regeneration in the sheep that lends itself to strict standardization and in which a number of substances can be tested within the same animal. To this end the caudal border of the ovine scapula was used as a consistent bed of mineralized tissue that provided sufficient room for a serial alignment of multiple experimental drill holes.

**Results:**

The findings show that for the sake of standardization, surgery should be restricted to the middle part of the caudal margin, an area at least 80 mm proximal from the Glenoid cavity, but not more than 140 mm away from it, in the adult female Land Merino sheep. A distance of 5 mm from the caudal margin should also be observed.

**Conclusions:**

This standardized model with defined uniform defects and defect sites results in predictable and reproducible bone regeneration processes. Defects are placed unilaterally in only one limb of the animal, avoiding morbidity in multiple limbs. The fact that five defects per animal can be evaluated is conducive to intra-animal comparisons and reduces the number of animals that have to be subject to experimentation.

## Background

Bone grafting takes place in over 100 000 procedures annually in the US [1]. Where autograft material is limited or inadequate, or when donor-site morbidity is to be avoided [2, 3], substitute materials are required. The market for bone graft substitute materials exceeds 2 billion dollars in a group of 10 major countries [4].

This need for bone graft substitutes also motivates the development of new methods to improve bone regeneration; increasingly, these novel treatment techniques include tissue engineering and cell-based approaches. Proof of concept in bone regeneration studies can only be shown with the help of animal models; no in vitro method can mimic the complexity of an in vivo environment sufficiently or predict clinical efficacy. Whereas initial screening and feasibility testing are popularly carried out in rodent models, large animal models whose bone regeneration is closer to the same processes in humans are essential to provide translational proof of concept.

The FDA, for example, often requires the testing of bone therapies in both a small and large animal model before accepting an agent for clinical trials [5, 6]. Rodent models cannot adequately mimic human bone regeneration for a number of reasons, among them a lack of cortical remodeling and the fact that cessation of growth occurs much later than in other mammals [7, 8]. The biomechanical conditions of human skeletal loading can obviously not be simulated in small animal models with their lower body mass. The mechanisms of bone regeneration depend on the size of the defect, because a mass transport of oxygen and nutrients, cell migration and vascular invasion into and the removal of degradation products out of the defect area are strongly influenced by the distances that have to be overcome [9, 10]. This constitutes a need to create large defects that can only be set in large animals [7]. Also the fact that the immune system of large animals is more similar to humans than that of rodents is especially important when the influence of immunogeneic substances and (allogeneic) cells on bone regeneration is studied [11–16].

Although many studies have been carried out already [17, 18], no final consensus on the standardization of large animal models has been reached so far. Factors that potentially influence the outcome like defect size, time points of evaluation or the species of test animal continue to be controversially discussed.

One important requirement is that the model should not be too rigid in its application. When new experimental needs and different indications arise it should be adaptable enough to meet these new demands. However a standardized animal model where the quality and quantity of bone regeneration and its ability to integrate bone substitute material are known in detail could be of great value. The most important factor to achieve this standardization is the fact that several defects can be evaluated in the same narrow anatomical region of the same individual animal. Uniform surgical technique is paramount to facilitate comparability in animal models and the exact location of a bone defect has a profound influence on the bone regeneration properties being tested. Hopefully the sum of this could lead to a wider acceptance of this model as a standard method in testing bone graft substitute materials and thereby improve comparability between studies. Standardization may be the key to moving systematically towards better bone regeneration therapies in the future.

In the following study, we aimed to establish an orthotopic model of bone regeneration in the sheep that allows stringent standardization and in which several test substances can be analyzed within the same animal. The products or substances that are about to be tested in the defects should be subjected to identical physiological influences by being implanted in the same narrow anatomical region where conditions are constant. We describe here the establishment of this standardized model from selection of animals to surgery to the histologic and morphometric evaluation as it has been proven in a recently published study on the preclinical testing of hydroxyapatite biomaterials [19].

## Results

All animals tolerated the surgery well, no lameness or other clinically relevant findings were seen after the immediate postoperative period and the animals returned to free pasture after sutures were removed 10 days after surgery.

All samples from all animals were available for CT analysis although two defects were not analyzed due to CT reconstruction problems. One defect site was not available for histological analysis because of faulty drilling.

### Anatomical and histological characteristics of the experimental site

**Fig. 1:**
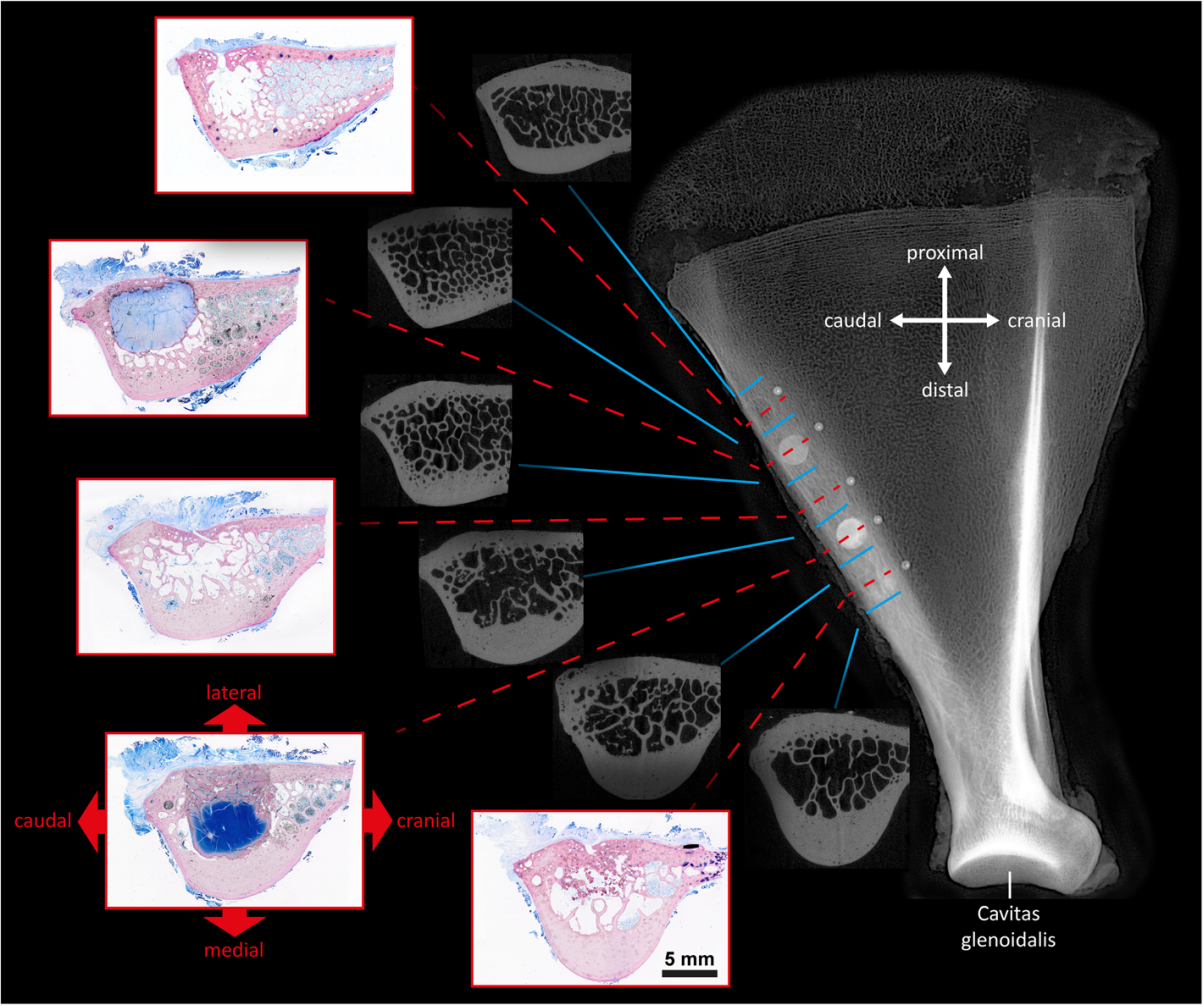
Anatomical and histological characteristics of the ovine scapula model. On the right a radiographic presentation of the scapula in lateral view with 5 drill holes in the caudal margin. On the left the histologic specimens through the center of the defects (red lines) filled with different bone substitute materials showing the regional differences from proximal to distal. In the center μCT images depicting the regions between the drill holes (blue lines). The medial bulge decreases in height from distal to proximal while its width increases

**Fig. 2:**
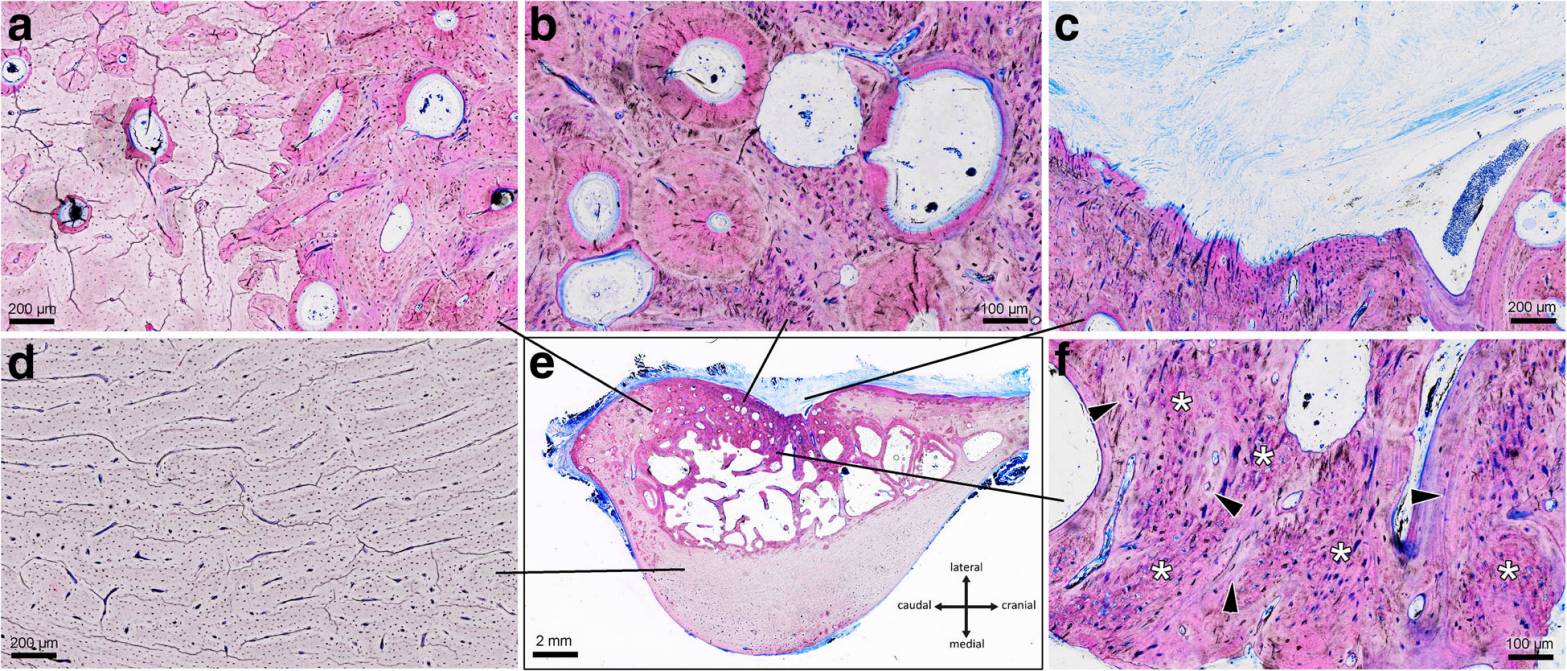
Histological presentation of the involved tissue types. An overview of the region is depicted in the lower center (black frame). The newly formed bone tissue inside of the drill hole is stained darker purple than the surrounding old bone tissue. Histological details in higher magnification are arranged around the overview: **a** The border of the drill hole, with old bone tissue on the left and darker newly formed bone tissue on the right is still visible. **b** Inside of the newly formed bone a large number of secondary osteons in all stages of formation are present. They are surrounded by the remnants of primary plexiform bone. **c** Parts of the drill hole are filled with fibrous soft tissue that penetrated the defect from the periosteal side. **d** The cortical bone of the medial bulge consists of plexiform bone. **e** Plexiform bone is also found inside of the defect. It consists of a network of woven bone (asterisks) on which parallel fibered bone (arrow heads) was laid down. This primary type of bone tissue is continuously replaced by the lamellar bone of secondary osteons. Micrographs of undecalcified thin ground sections stained with Levai-Laczko dye

**Fig. 3:**
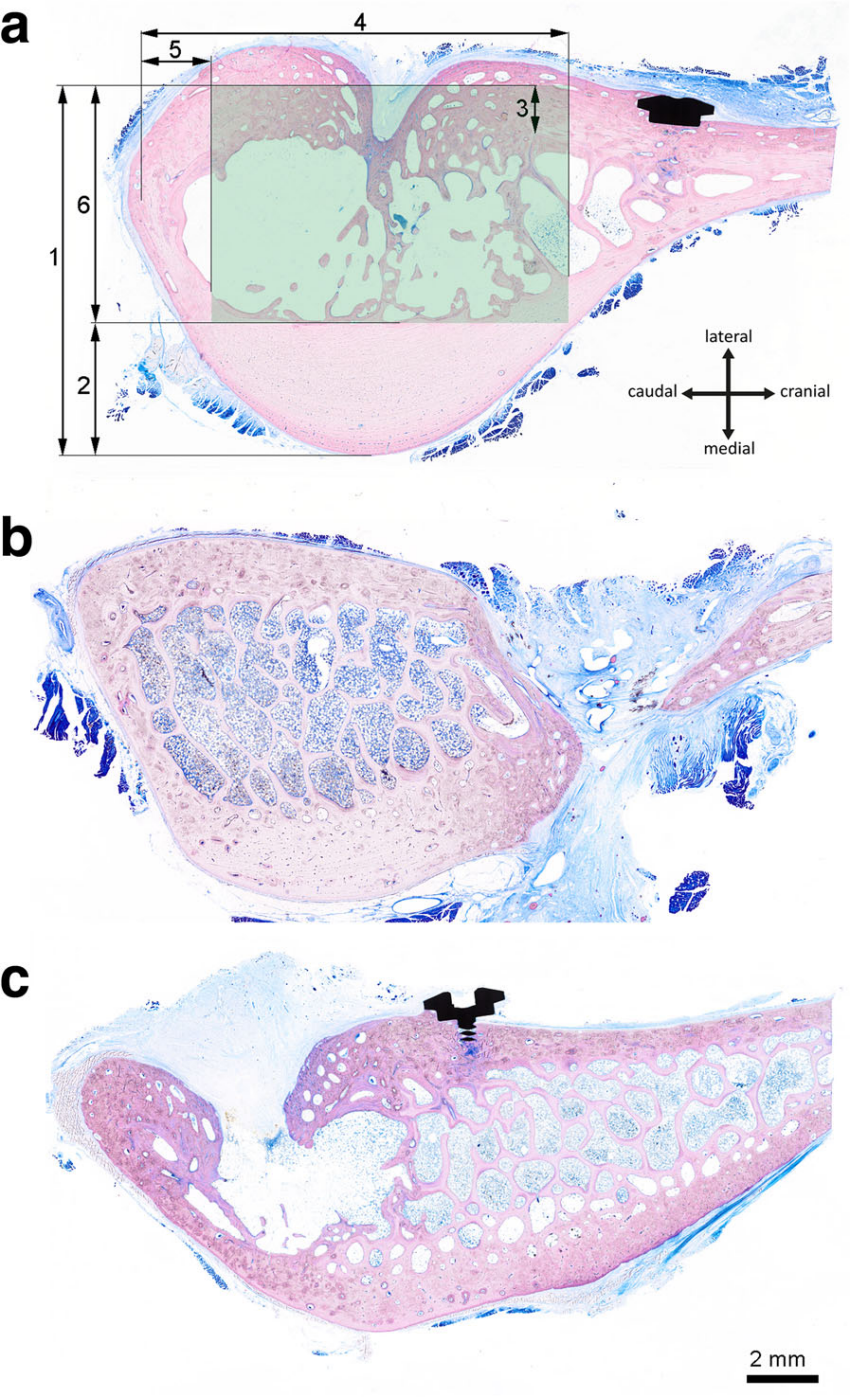
**a** Definition of the measurements for the characterisation of the anatomical properties of the experimental site in the shoulder blade of sheep: Total height of the medial bulge (1), cortical thickness of the medial wall (2), cortical thickness of the lateral wall (3), width of the medial bulge (4), minimal necessary distance from caudal margin (5), height of experimental space (6). The experimental space in which the defect should be placed to achieve controlled and reliable results is marked by a green inlay. **b** Cranially displaced drill hole breached both cortical walls. **c** Caudally displaced drill hole located too close to the caudal cortical wall

**Fig. 4:**
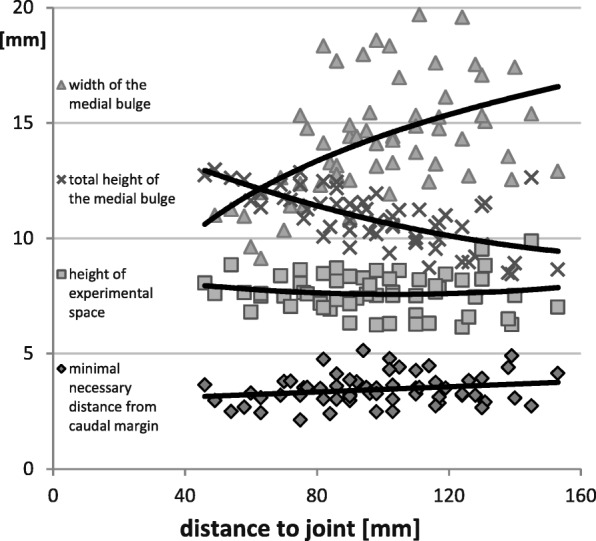
Variation of the dimensions of the test region dependent on the distance from the glenoid cavity of the ovine scapula. (Definition of measurements see Fig. 3)

The caudal border of the ovine scapula is a thick bony structure that provides a bone volume that is sufficient for placing several drill hole defects in a row parallel to the caudal margin (Fig. 1). While the main body of the shoulder blade is flat and thin, the caudal border shows a bulge or thickening on the medial aspect. This bulge or torus is predominantly semi-circular in cross-section and is surrounded by cortical bone of the plexiform type (Fig. 2d), while the center is filled with lamellar cancellous bone and fatty marrow. The overall height of the semi-circular bulge (Fig. 3a-1) is on average 10.9 +/− 1.2 mm and its width (Figs. 3a-4) is 16.9 +/− 2.5 mm. There are topographic differences: closer to the Glenoid cavity the ridge is higher and narrower, but it flattens out in the proximal direction, i.e. it becomes lower and wider (Figs. 1, 4).

The cortical wall of the bulge is thickest on the medial face (Fig. 3a-2) with a mean value of 3.2 +/− 0.9 mm. Again, the dimensions show a gradient, because the bone is thicker close to the joint and thinner farther away from it. In the cortical bone of the lateral face (Fig. 3a-3) where the defects are placed, no such change in dimension can be observed. It shows an almost constant thickness of about 1.3 +/− 0.3 mm over the whole area. A demarcation of the bulge in the cranial direction is difficult to make, as it gradually becomes thinner. In some cases, the medial and lateral cortical plate fuse with one another, forming one unified cortical plate (Fig. 3a). In other cases, the two plates run parallel to each other, separated by a thin layer of cancellous bone (Fig. 3c). For the purposes of this study, the cranial border of the bulge was per definition placed at the point where the lateral cortical bone wall together plus the marrow space were at least 5 mm high (Fig. 3a-4). This demarcates the area where the drill holes can be optimally placed and constitutes the relevant experimental space available for the placement of the drill hole and the test substances (green area in Fig. 3a). This experimental space shows little variation in its height in the proximodistal direction (Fig. 4) while its width increases in the proximal direction.

**Fig. 5:**
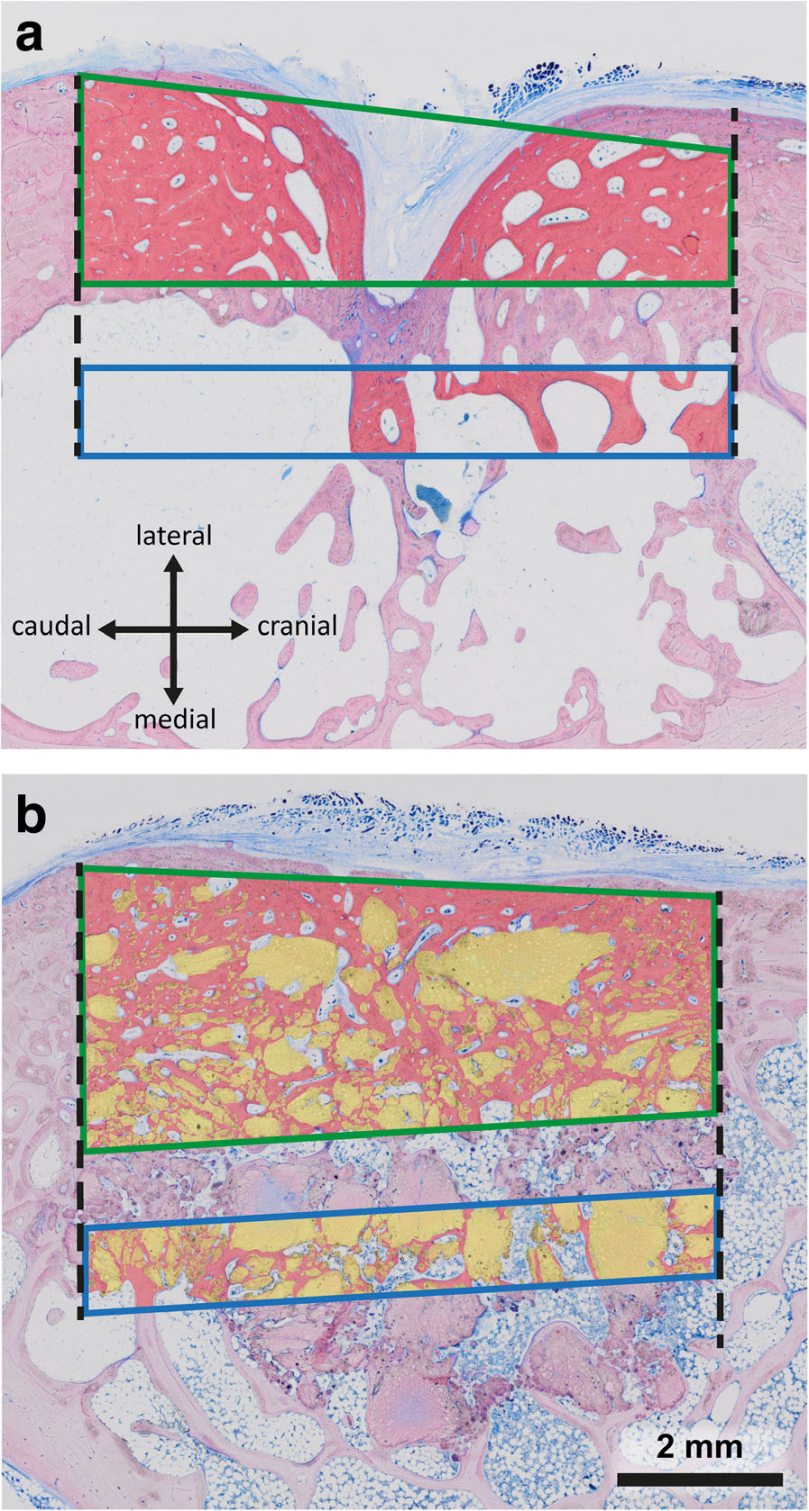
Presentation of the regions of interest in the caudal border of the ovine scapula used for histomorphometric evaluation. The cortical region of interest inside of the defect is surrounded by a green frame while the medullary region of interest is depicted in blue. The margins of the drill hole are presented as a black dashed line. Newly formed bone is red and bone substitute materials yellow. **a** empty control defect; **b** defect filled bone substitute material

The minimum necessary distance of the drill hole to the caudal margin (Fig. 3a-5) was measured on histological specimens. This distance was important to guarantee a minimal height of the experimental space of 5 mm and to securely place the defect in the marrow space without impinging on the caudal cortical bone. On average this minimal necessary distance from the caudal margin was 3.4 ± 0.7 mm (Fig. 4). The absolute largest value was 5.1 mm.

### Histologic and histomorphometric healing characteristics

Not all of the defects had fully healed after 6 months. In the empty control group only 27% of the drill holes and 17% of the autologous bone group were completely bridged. Discontinuities in the cortical region left deep depressions that penetrated into the marrow space (Figs. 2c and 5a).

The bone tissue that filled the drill holes was a mixture of lamellar bone in the form of secondary osteons and interspersed remnants of plexiform bone (Fig. 2b). Plexiform bone, which consists of woven bone and parallel fibered bone (Fig. 2e), is formed in the early stages of bone healing [20, 21]. It initially fills the gaps and stabilizes the defect. After mechanical stability is achieved, this primary bone is resorbed by osteoclasts and replaced by more mature secondary lamellar bone, formed by osteoblasts. At 6 months, this process of remodeling had already progressed far in our model. In most cases, more than half of the primary plexiform bone in the cortical region had already been replaced by secondary osteons. This process of remodeling was still going on at a very high rate, as proven by the presence of many large secondary osteons in all active stages of formation (Fig. 2b). In the cancellous region of the defect, trabecular bone filled the space and was also a mixture of primary plexiform and secondary lamellar bone, but remodeling activity was far lower than in the cortex. Only a few hemiosteonal remodeling sites could be observed on the surface of the trabeculae. In the autochthonous bone surrounding the drill hole, pre-existing trabeculae were compacted and thickened by layers of bone that had been added posttraumatically. A similar effect was seen at the periosteal surface surrounding the defects, where often large quantities of bone tissue had been laid down.

After 12 months, the findings were not remarkably different. In the empty control group 33% showed continuous bony bridging while in the autologous bone group the rate was 25%. Histologically, the tendencies detectable at 6 months had simply continued and progressed. More, but not all, of the primary bone had been converted by remodeling into secondary osteons. The most striking difference was the lower rate of ongoing remodeling. Far fewer active remodeling sites were seen than at the 6 month time point.

These qualitative histologic findings were not significantly different between drill holes filled with autologous bone and those left completely empty. Autologous bone graft had obviously been resorbed rapidly; only small traces of it were still detectable after 6 months.

### Histomorphometry

After 6 months, the mean percentage of newly formed bone (nBV/TV) in the cortical region was 54.39 ± 21.71% for the empty control while it was 60.36 ± 18.34% in the autologous bone group, i.e. the defects were about half filled, with a large degree of variation. In the medullary areas, nBV/TV for the empty control was 28.52 ± 14.20% and that for autologous bone 18.34 ± 13.82%. Bone regeneration was only about half as strong as in the cortical environment. There were no significant differences between the two treatment groups.

After twelve months, the percentage of newly formed bone in the cortical region was not greatly different from that at 6 months. The drill hole in the empty control group was filled to 56.34 ± 25.92% with new bone and that of the autologous bone group to 59.83 ± 27.98%. The situation in the medullary space was not as unambiguous. The empty control group showed higher values for nBV/TV of 40.56 ± 15.85% while in the autologous bone group they were with 32.41 ± 13. Again, there were no significant differences between the treatment groups.

To characterize the completeness of bone regeneration in a cortical defect, it is important to measure the fraction of the area that was not filled with bone, but with soft tissues that invaded from the periosteal region and impaired bone formation. The percentage of these soft tissues in areas were only cortical bone should be was 35.06 ± 25:67% for the empty control and 29.72 ± 22.55% for autologous bone after 6 months. After a period of 12 months, the values were 37.26 ± 30.69% and 32.67 ± 33.39% respectively. Variation was conspicuously high.

These results for nBV/TV have been used in the publication by Hruschka et al. [19] as control groups in comparison to several bone substitute materials but were not described in greater detail. They are presented here more extensively in order to illustrate the quantitative healing properties of the experimental model.

### Description of technical problems with the model

The exact placement of the drill holes proved to be the most severe problem. The planned ideal defect, which should cut through the lateral cortical wall and then penetrate the medullary space with bone marrow surrounding it on all sides (Fig. 3a) could not always be established in the in vivo scenario. In some cases the drilling was placed too close to the caudal margin and the caudal cortical bone was penetrated leading to stronger bone formation due to the greater regenerative potential of this cortical region (see histomorphometric results). A drill hole placed too far caudally was subjected to disproportionately high bone formation (Fig. 3c). If the drill hole is located too far cranially, there is a risk of cutting through the entire bone thickness, which is thinner in these regions (Fig. 3b).

The titanium pins marking the centre of the defects proved to be unnecessary. Even after long observation periods, the empty drill holes were clearly visible on x-rays. Degradation of bone graft substitute material was easily followed over periods up to one year. A critical size model could not be established. Bone growth after one year in the empty defects was much higher than the 10% that stipulates a critical size defect [22] but healing was severely impaired.

## Discussion

In the adult sheep a 60 mm long area of the caudal border of the scapula shows a bone architecture and quality constant enough for a standardized serial alignment of multiple test drill holes. To uphold and improve standardization, surgery should be restricted to the middle part of the caudal margin because of the regional differences in the height and width of the bulge mentioned above. A reasonable recommendation is to use an area beginning at least 80 mm from the Glenoid cavity, but not more than 140 mm away from it (Fig. 4). The narrower the chosen region is, the more similar the anatomical and histological characteristics will be. Nevertheless, there should be at least 5 mm distance from the margins of one drill hole to the next, so that the healing processes of the individual defects will not interfere with one another. These measurements apply to the adult female Land Merino breed and will differ in other breeds and in male sheep.

Precision operating technique is needed in this model and is perhaps the most challenging aspect with the greatest influence on standardization. That a true critical size defect is not possible in this anatomical site is, indeed, an admitted weakness.

The immediate milieu and environment of drill hole test sites consists of well vascularized muscle tissue on one side and bone marrow on the other side providing a plethora of progenitor cells and factors necessary for defect healing. Because of this, the region facilitates substantial new bone growth and, in longer observation periods, allows the evaluation of remodeling and degradation of bone substitutes. The sharp dissection of the muscle tissue adjacent to the scapula test area also does, to a certain extent, mimic the soft tissue damage seen in the vicinity of a typical traumatic wound/defect.

Sheep are compliant and docile animals [23] and are a less controversial test subject in the public opinion than, for example, the dog. The body weight of sheep and the size of its long bones are roughly similar to those of humans. Their remodeling rate, lamellar bone structure and primary bone healing characteristics are similar to humans [11]. Monitoring and delivery techniques can be the same as those used in humans. The ability to use young, aged or ovariectomized ewes as well as male animals means that the influences of age, sex and pathologies such as osteopenia/osteoporosis can be simulated in sheep models. When biomechanical testing is important, large animals such as the sheep have an advantage over rodent models [24] as they more readily approximate human anatomy.

A number of models have been developed, also in the sheep and also utilizing drill hole defects, to test bone graft substitutes [12, 23–25], some placing the drill holes in the long bones of the extremities, others using a combination of femur drill holes and a slot defect in the tibia [26–28]. The comparison of different variants or different dosages of bioactive substances is best achieved when comparisons of all can be made in a single animal. Nuss et al. and others [17, 25] proposed a sheep model with 8 bilateral drill hole sites in both the femur and humerus. Van der Pol achieved a higher degree of standardization [29] by using customized drilling jigs but in sites in two different bones (the tibia and femur). Gisep et al. tested nonloaded bone regeneration in a drill hole model combined with a loaded tibia slot defect [27]. The use of different anatomical bone sites are not, however, comparable with regards to loading boundaries and amount and quality of new bone growth and bone densities [24].

The site of a bone defect has a large influence on its properties of regeneration. The density of bone in various anatomic sites varies widely [24]. When multiple defects are drilled in the epiphysis, their differing proximity to the metaphysis can directly influence growth and tissue characteristics. Defects placed in the metaphysis and the neighboring diaphysis differ starkly in new bone growth [30]. Bone regeneration is site specific; in our model, all graft samples and controls are placed in the same osteologically homogenous anatomical region. Different bone sites are also influenced by the type of tissue in its immediate vicinity (i.e. muscle) and may suffer from lack of uniform bone growth environments.

The use of bilateral defects, in which limbs on both sides of the animal are affected, are discouraged due to animal welfare reasons [7].

A model in which all test substances can be implanted in a single animal can keep animal numbers down [31]. The assessment of multiple defects in one animal provides for control of variation between animals and thus limits the number of animals needed [7]. Indeed, the main advantage of this model is the possibility of implanting 5 different test items or allowing 5 treatment groups in a single large animal while refraining from setting a bilateral defect.

This model allows for implantation biocompatibility testing of new bone graft substitutes as well as efficacy testing, although short term evaluations maybe more efficient in, or in combination with, a rodent model. Short term observation periods would allow for the tracking of inflammatory reactions, osteofibrosis or osteonecrosis. The model lends itself particularly well to following and comparing the degradation characteristics of bone graft substitutes and their replacement with new bone over longer periods of time.

Clearly, no animal model matches human physiology perfectly. Sheep do tend to have higher trabecular bone densities than humans, depending on site [32, 33]. Although some breeds cycle all year round, sheep are seasonal breeders that have varying oestrogen levels over the course of the year and this must be taken into account during the study design phase and season of the year of any interventions reported [18, 34].

This model also allows for the use of autologous graft that is comparable to the cancellous graft applied in clinical human use – something that is not possible in rodents in which typically only bone marrow with cortical bone and periosteum is available for grafting and in which osteogenic colony forming units in grafts are much lower than in humans [7].

Aside from the practicalities and the mechanical factors that speak for the use of large animals in bone regenerative medicine, there are other biological reasons as well. An example of this is the difference in bone regeneration between small animals and large animals as can be seen in the fact that calcium phosphate scaffolds rarely show osteoinduction in rodent models while successfully inducing bone growth in large animals [35]. Also the immune system of large animals is more similar to humans than that of rodents [11–16].

One of the largest obstacles in the study of regenerative medicine is the host/graft reaction against human cells in animal models. No immunodeficient sheep model is available for cell transplantation, although protocols of sheep immunosuppression have been developed [36]. Looking towards the future, induced pluripotent stem cells have been recently produced from sheep cells [37, 38] and may find their way into preclinical testing methods.

## Conclusion

We have presented here a large animal model for the study of bone regeneration that is relatively easy to perform and provides a strictly standardized testing assay from surgery to quantitative evaluation. It has defined uniform defects and defect sites with uniform regeneration and allows up to 5 defects per animal, conducive to intra-animal comparisons and to reducing animal numbers. Defects are placed unilaterally in only one limb of the animal, avoiding the morbidity associated with the use of multiple limbs seen in other models.

## Methods

### Animals

This study was approved by the local ethics committee (“Thüringer Landesamt für Verbraucherschutz”) and was consistent with the Guide for the Care and Use of Laboratory Animals of the National Institute of Health (revised 2011) and the European directive 2010/63/EU.

The model was tested in a study by Hruschka et al. [19] using 24 adult (aged 2–3 years) female Land Merino sheep. Empty ewes which had experienced at least one lactation period were purchased by the research institute from an agricultural company prior to the study. The animals were fed with hay and allowed to drink water ad libitum and housed in a free stall barn in the immediate postoperative period, then transferred to free ranging pasture.

General anaesthesia was induced by an intravenous administration of xylazine (Rompun, Bayer GmbH), diazepam (Gewacalm, Takeda GmbH) and ketamine (Ketamidor, Richter pharma AG) (0.1 mg/kg, 0.1 mg/kg, 4.4 mg/kg). After induction, the animals were intubated and anesthesia was maintained with isoflurane at 1.0–1.5 vol% and oxygen. The animals received both a pre-emptive analgesic, carprofen (Rimadyl, Pfizer Inc.; 1.4 mg/ kg intravenous) and a systemic antibiotic (Veracin-Compositum, Albrecht GmbH, 3 ml/50 kg intramuscular). Haematological and parasitological examinations were performed prior to the study and animals were operated on in late autumn.

### Surgery

The animal was laid in lateral recumbency. The region of the left shoulder blade was clipped, shaved and disinfected and the animal was draped. A longitudinal skin incision approximately 15 cm long and parallel to the caudal border of the left scapula was made using the acromion and caudal angle of the scapula as landmarks. The caudal margin of the scapula was exposed by sharp dissection of the musculature. All soft tissue was separated from the bone with a periosteal elevator. Using a trephine drill, five defects with a diameter and depth of 8 mm were created, leaving a 5 mm bony bridge in between each defect. The defects were irrigated with saline solution during drilling. The longitudinal axis of the drill holes was oriented perpendicular to the lateral face of the scapula and included the lateral cortical layer and the trabecular layer down to the medial corticalis (Fig. 1).

The cylinders from the drill holes were preserved for autologous bone grafting. Titanium pins (3 mm long, 0.7 mm in diameter) were placed close to the defects to mark their exact location. The five drill hole defects were randomized to either an empty control, autologous graft or test article group. Autologous cancellous material was mixed together as a rough paste before implantation. The surgical incision was closed in multiple layers using absorbable Monocryl 3–0 (Ethicon Inc.) suture material with the skin closure using everting horizontal mattress sutures and nonabsorbable material. The animals received carprofen subcutaneously for five days post-operatively.

Animals were euthanised under general anaesthesia as described above. T61, a commercial euthanasia solution based on embutramide, mebozonium, iodide and tetracaine hydrochloride (Merck Animal Health, Munich, Germany), was administered by a trained veterinarian by slow intravenous injection at a dosage of 0.1 ml/kg body weight. Sampling was carried out at 6 months and 12 months after treatment.

### Histology and sample preparation

The left shoulder blades were removed from the animals and fixed in buffered formalin. An overview radiograph was produced of each sample to ascertain the exact location of the augmentation sites. Blocks containing each one of the defects were cut using a diamond coated band saw (EXAKT Apparatebau, Norderstedt, Germany) separating them precisely in between the drill holes as visible on the radiograph (Fig. 1). These blocks were scanned after 14 days of fixation with a SCANCO μCT 50 (SCANCO Medical AG, Bruttisellen, Switzerland) micro-CT (μ CT) at 90 kV/200 μA with an isotropic resolution of 17.2 μm and an integration time of 500 ms. The scans were exported as 16 bit DICOM image stacks. Three-dimensional reconstruction of the data was performed using the visualization software Amira 6.2 (Thermo Fisher Scientific, Waltham, USA).

After μCT scanning, blocks were dehydrated and embedded in plastic (Technovit 7200 VLC + BPO; Kulzer & Co., Wehrheim, Germany). Undecalcified, thin ground sections were made using a method established by Donath [39]. These histologic specimens were stained with Levai-Laczko dye, a variant of the Giemsa dye that allows to distinguish reliably between old bone, new bone and bone substitute materials [40].

This model allows qualitative and descriptive histological evaluations of bone regeneration as well as a histomorphometric analysis. Histologic thin ground sections were segmented histomorphometrically using Definiens Developer XD 2.1 (Definiens AG, Munich, Germany) and corrected manually using Photoshop CS 4 (Adobe Systems Inc., San Jose, CA, USA). The corrected images were then measured in Definiens Developer XD 2.1.

Two specific regions of interest were defined to allow a differentiated evaluation of bone growth and test substance degradation. The cortical region of interest was defined as the area between the boundaries of the drill hole inside the cortical bone and the periosteal and endosteal margins of the neighboring bone. A medullary region of interest was set at a location 1 mm beneath the cortical region of interest and had a height of 1 mm (Fig. 5). This medullary region of interest included the marrow space within the whole breadth of the drill hole. The corrected images then were measured using the Definiens software. Examples of evaluation parameters that can be measured include: areas of newly formed bone, areas of soft tissue, amount of residue substitute material and the complete region of interest (tissue volume). Also, the sizes of individual graft substitute particle agglomerates and their number can be determined. From these primary measurements the following parameters can be calculated: The percentage of bone substitute material in the regions of interest, the average size of particle agglomerates in mm^2^, the number of bone substitute particle agglomerates per mm^2^, the percentage of newly formed bone in the regions of interest (BV/TV), the percentage of the composite of newly formed bone plus bone substitute material, the percentage of the region of interest filled with soft tissue that had grown from the periosteal region into the defect area (Periosteal void volume). An example of the model that used these parameters of evaluation has been published [19].

For the purposes of this study, only the bone healing of the empty control group and the autologous bone group were histomorphometrically characterized. These two treatment regimens are commonly used as a control and in comparison with newly developed test substances. The amount of newly formed bone tissue (nBV) was divided by the size of the complete region of interest (TV), resulting in the percentage of new bone tissue inside of the region of interest (nBV/TV).

### Statistical evaluation

Descriptive statistics were performed calculating means and standard deviations. Multiple linear mixed models for all independent variables were constructed, where treatment was used as an independent variable and animal ID as a random factor. Tukey-type post-hoc tests were calculated for treatment effect [19].

All computations were done using R version 3.2.3 [41].

### Micro-CT

We scanned samples at 90 kV with an isotropic resolution of 17.2 μm. We evaluated the closure of defects and categorised them either as “non-bridging” (leaving a continuous opening in the cortical bone which allowed communication of the medullary cavity with the periosteal area) or “bridging” when the continuity of the cortical bone was completely restored.

## Abbreviations

BPO: Benzoyl peroxide
nBV/TV: New bone volume per tissue volume
μCT: Microcomputed tomography
